# Who seeks primary care for musculoskeletal disorders (MSDs) with physicians prescribing homeopathic and other complementary medicine? Results from the EPI3-LASER survey in France

**DOI:** 10.1186/1471-2474-12-21

**Published:** 2011-01-19

**Authors:** Michel Rossignol, Bernard Bégaud, Bernard Avouac, France Lert, Frédéric Rouillon, Jacques Bénichou, Jacques Massol, Gérard Duru, Anne-Marie Magnier, Didier Guillemot, Lamiae Grimaldi-Bensouda, Lucien Abenhaim

**Affiliations:** 1 Department of Epidemiology, Biostatistics and Occupational Health, McGill University, Canada and LA-SER Centre for Risk Research, Montreal, Canada; 2 INSERM U657, Université Bordeaux2, Bordeaux, France; 3 LA-SER, Paris, France; 4 INSERM, Centre for Epidemiology and Population Health, Villejuif, France; 5 Centre Hospitalier Sainte-Anne, Université Paris V René Descartes, Paris, France; 6 INSERM U657 Pharmacoepidemiology and evaluation of the impact of health products on human health, France, and Department of Biostatistics, University Hospital of Rouen, Rouen, France; 7 UFR de Médecine, Université de Franche Comté, Besançon, France; 8 CNRS (French National Center for Scientific Research), Université Claude Bernard, Lyon, France; 9 Faculté de médecine Université Pierre et Marie Curie, Paris, France; 10 Institut Pasteur and Université Paris-Ile de France Ouest, Paris, France; 11 Equipe d'acceuil Pharmacoépidémiologie et maladies infectieuses, Institut Pasteur, and LA-SER, Paris Cochin santé, France; 12 Department of Epidemiology, London School of Hygiene & Tropical Medicine, London, U.K., and LA-SER, London, U.K

## Abstract

**Background:**

There is a paucity of information describing patients with musculoskeletal disorders (MSDs) using complementary and alternative medicines (CAMs) and almost none distinguishing homeopathy from other CAMs. The objective of this study was to describe and compare patients with MSDs who consulted primary care physicians, either certified homeopaths (Ho) or regular prescribers of CAMs in a mixed practice (Mx), to those consulting physicians who strictly practice conventional medicine (CM), with regard to the severity of their MSD expressed as chronicity, co-morbidity and quality of life (QOL).

**Methods:**

The EPI3-LASER study was a nationwide observational survey of a representative sample of general practitioners and their patients in France. The sampling strategy ensured a sufficient number of GPs in each of the three groups to allow comparison of their patients. Patients completed a questionnaire on socio-demographics, lifestyle and QOL using the Short Form 12 (SF-12) questionnaire. Chronicity of MSDs was defined as more than twelve weeks duration of the current episode. Diagnoses and co-morbidities were recorded by the physician.

**Results:**

A total of 825 GPs included 1,692 MSD patients (predominantly back pain and osteoarthritis) were included, 21.6% in the CM group, 32.4% Ho and 45.9% Mx. Patients in the Ho group had more often a chronic MSD (62.1%) than the CM (48.6%) or Mx (50.3%) groups, a result that was statistically significant after controlling for patients' characteristics (Odds ratio = 1.43; 95% confidence interval (CI): 1.07 - 1.89). Patients seen by homeopaths or mixed practice physicians who were not the regular treating physician, had more often a chronic MSD than those seen in conventional medicine (Odds ratios were1.75; 95% CI: 1.22 - 2.50 and 1.48; 95% CI: 1.06 - 2.12, respectively). Otherwise patients in the three groups did not differ for co-morbidities and QOL.

**Conclusion:**

MSD patients consulting primary care physicians who prescribed homeopathy and CAMs differed from those seen in conventional medicine. Chronic MSD patients represented a greater proportion of the clientele in physicians offering alternatives to conventional medicine. In addition, these physicians treated chronic patients as consulting rather than regular treating physicians, with potentially important impacts upon professional health care practices and organisation.

## Background

Physicians in primary care play a central role in the management of musculoskeletal disorders (MSDs). This group of health problems also represents a leading reason of recourse to complementary and alternative medicines (CAM) including homeopathy. There is a paucity of information describing MSD patients using CAMs and almost none distinguishing homeopathy from other forms of CAM. In France, homeopathy is the most frequently used CAM and is prescribed only by physicians, typically general practitioners (GP). In addition, homeopathy is reimbursed by the French National Health Insurance which allowed a fair comparison of patients who seek care with physicians who prescribe and don't prescribe homeopathy and CAMs. The objective of this study was to describe and compare patients with MSDs consulting primary care physicians, either certified homeopaths (Ho) or regular prescribers of CAMS in a mixed practice (Mx), to those consulting physicians who practice strictly conventional medicine (CM), with regard to the severity of their MSD expressed as chronicity, co-morbidity and quality of life (QOL).

## Methods

### Study design and population

This analysis used data collected from the EPI3-LASER nationwide observational survey of a representative sample of general practitioners and their patients, conducted in France between March 2007 and July 2008. Its aims were to assess the burden of disease in general practice, considering physician and patients characteristics and co-morbidities with a specific focus on health-related QOL. Study subjects were drawn from a two-stage sampling. First, general practitioners (GPs) were randomly selected from the French national directory of physicians and invited to participate. GPs sampling was stratified according to their declaration of CAM utilisation categorised in three groups, strict conventional medicine practitioners (CM) who declared themselves never or rarely using homeopathy or CAMs, physicians declaring using CAMs regularly in a mixed practice (Mx), and registered homeopaths who are GPs specialised in homeopathy (Ho). Physicians were classified in one of those three categories after they had agreed to participate to the survey by answering a short telephone questionnaire designed to that effect. As physicians in all three groups were certified professionals, their practice was based on conventional medicine. The Ho group differed because the physicians were also certified homeopaths with a clear orientation toward this type of CAM. The Mx group however could not be labelled specifically as they only declared their use of CAMs but did not indicate the type or any specific preferences.

Sampling of physicians continued until Mx and Ho GPs were over-sampled compared to CM GPs with ratios respectively of 2:1 and 3:2. This was done in order to account for the variety of practices, especially in the Mx group. The second stage of sampling consisted of randomly selecting a one-day of consultation for each participating physician to survey all patients attending the practice on that specific day. All patients were eligible for inclusion at the exception of those whose health status or literacy level did not allow responding to a self-administered questionnaire.

### Data collection

Consenting patients completed a self-administered questionnaire that included information on age, sex, education, employment status and occupation, hospitalisation and medical visits in the previous twelve months, smoking, alcohol intake, physical activity, height and weight, and health related QOL assessed by the Short Form 12 (SF-12) questionnaire [1]. Patients were also asked to declare if the physician consulted that day was their regular treating physician or not. In France all citizens are required to identify a physician for their regular health care. In this study, the physicians' role was labelled 'treating' or 'consulting' based on patients' responses. GPs completed a medical questionnaire including the main reason for consultation and up to five other diagnoses present that day and for each, the duration of the health problem in its current episode. GPs then recorded their prescriptions that day for diagnostic tests, drugs and referrals. Diagnoses were coded by a trained archivist using the 9^th ^revision of the International Classification of Diseases.

for this analysis, adult patients 18 years and older with a MSD as their main reason for consultation, were selected from the general survey. MSDs included spinal disorders (SD) with ICD codes 720 to 724, osteoarthritis and tendonitis of the upper or lower limb, with ICD codes 715, 719, 729, 726-728, 782. Patients with a diagnosis of infectious, neoplastic or specific inflammatory (such as rheumatoid arthritis or Lupus) joint disease as their main reason for consultation were excluded from the analyses. MSDs were classified as non-chronic or chronic using a twelve-week (three months) cut-off for duration of symptoms at inclusion in accordance with consensus recommendations for research on MSDs [2]. Co-morbidity was defined as the presence of at least one diagnosis other than the principal reason of consultation at the recruitment visit. Co-morbidities were categorised as MSD (other than the main reason for consultation), cardiovascular or respiratory, anxiety or depressive disorders, sleeping disorders and digestive.

### Statistical analysis

Patient non-participation was accounted for by performing a calibration of the sample using the CALMAR procedure on all variables that were collected from all eligible patients (sex, age, length of time attending the GPs' practice, type of health insurance and main reason for consultation) [3]. Multiple logistic regression analyses were used to compare patients in the Ho and Mx groups to the CM group for categorical variables and adjusted for all variables in Table 1 and 2 for potential confounding. Mean scores of the SF-12 mental and physical scales were adjusted for sex, age and co-morbidities using the analysis of covariance. All analyses were performed using SAS version 9.1.

**Table 1:** Socio-demographic characteristics of patients consulting for a MSD in primary care (N = 1692)

	Type of primary care provider		
	Conventional medicine	Mixed	Homeopathic
	N = 366	N = 778	N = 548
**Sex **(% Females)	59.0	64.2	76.9*

**Age categories**			
18-39	21.7	19.6	13.9*
40-59	35.8	39.2	37.3*
60 and over	42.4	41.2	48.8*

**Employment status**			
(%) Employed	47.5	44.9	42.7
Unemployed	2.6	4.3	2.0
Home maker	2.9	5.1	3.2
Student	2.4	1.5	1.5
Retired	44.6	44.1	50.5

**Educational level**			
(%) Secondary school or more	39.4	35.4	45.9*

**Familial status **(%)			
Living with a spouse	66.7	67.7	68.8
Living with children	36.8	40.3	36.4

**Body Mass Index**			
<25	46.3	48.8	59.1*
25-30	36.9	32.8	31.1*
30 and over	16.8	18.5	9.6*

**Tobacco consumption**			
(%) Non smoker	48.1	55.5	62.1*
Past smoker	25.7	24.1	23.1*
Current smoker	26.2	20.4	14.7*

**Alcohol Consumption **(%)			
Never	33.1	32.6	28.8
Sometimes	51.4	55.4	57.4
Daily	15.5	12.0	13.8

**Physical exercise**			
(%) 0-30 minutes per day	59.5	60.9	66.1
31 minutes and over	40.5	39.1	33.9

* Difference with conventional medicine category statistically significant (p ≤ 0.05) in logistic regression including all variables.

The study was approved by the French National Data-Protection Commission (CNIL) and the French National Council of Physicians (CNOM). Participating physicians received compensation fees for recruiting but not patients.

## Results

### Patients' characteristics

Eight hundred and twenty five physicians agreed to participate in the study. Their geographical distribution covered the 22 regions of France. Their median age was 52 years, 20% were female, 52% worked in solo practice and 78% practiced fee-for-service in addition to the general health insurance regime which corresponds to national statistics on medical manpower [4]. Of the 10,803 patients identified as potential participants, 2,151 (20%) declined participation and 93 were excluded because of missing information leaving a final sample of 8,559, of whom 1,692 (20%) had a MSD. Characteristics of patients by type of medical practice are shown in Table 1. Patients in the Ho group were more often older non-smoking females with higher education and a lower body mass index than in the CM group. Patients in the Mx group did not differ from those in the CM group.

### Type and severity of MSDs

Characteristics of MSDs by type of primary care practice are shown in Table 2. The mix of spinal (low-back pain and other back problems) and non-spinal MSDs was similar between groups with spinal representing over one third of the reasons for consultation.

**Table 2:** Characteristics of musculoskeletal disorders (MSD) in three types of primary care practice (N = 1,692)

	Type of primary care practice		
	Conventional Medicine	Mixed	Homeopathic
	(N = 366)	(N = 778)	(N = 548)
MSD site (%)			
Spinal	37.7	41.2	35.9
Non-spinal	62.3	58.8	64.1
Chronicity (%)			
>12 weeks	48.6	50.3	62.1
Physician role^1 ^(%)			
Treating	84.0	81.8	54.1
Consulting	16.0	18.2	45.9

Adjusted Odds ratios^2^			
Risk of chronicity (vs. conventional medicine)	1 (--)	0.98 (0.77 - 1.26)	1.43 (1.07 - 1.89)
Risk of chronicity when Consulting (vs. Treating)	1.12 (0.63 - 1.99)	1.48 (1.06 - 2.12)	1.75 (1.22 - 2.50)

1. Role of the physician as declared by the patient; in France every citizen has to choose a physician as their regular treating physician (treating), physicians who are not the regular treating physician are called here consulting physicians.

2. Logistic regression models controlling for all variables in Table 1 and 2.

The first indicator of severity of MSD, chronicity, was more often observed in homeopathic practice, with 62.1% lasting for more than twelve weeks at the time of the survey, *versus *48.6% in the CM group, a difference which was statistically significant in multivariate analysis with an Odds ratio of 1.43 (95% confidence interval (CI): 1.07 - 1.89). At the same time, homeopaths were less often declared as the regular treating physician by their patients with 54.1% compared with 84.0% in the CM practice group. An interaction was observed between chronicity of the MSD and not being the regular treating physician (consulting). An excess risk of chronicity of 75% and 48% respectively was observed in MSD patients in the Ho and Mx groups when the physician was a consultant compared to being the regular treating physician (Odds ratios respectively 1.75, (95% CI : 1.22 - 2.50 and 1.48, 95%CI: 1.06 - 2.12).

The second indicator of severity of MSD was the frequency of co-morbidities (Table 3). Proportions of patients with any type of co-morbidities were very similar between the three groups of patients. MSDs as secondary diagnoses (in addition the MSD main diagnosis) were relatively frequent with 13.1% to 15.7% across the three groups of patients. Similar proportions were observed for anxiety and depressive disorders (12.5% to 14.6%). Sleeping and digestive disorders were less frequent co-morbidities and did not show important differences between types of physicians' practice.

**Table 3:** Co-morbidities in patients with musculoskeletal disorders (MSD) in three types of primary care practice (N = 1,692)

	Type of primary care practice		
Co-morbidities present at the medical visit	Conventional Medicine	Mixed	Homeopathic
	N = 366	N = 778	N = 548
**At least one MSD co-morbidity **(%)	13.8	13.1	15.7

**At least one other co-morbidity **(%)	72.7	73.3	74.4

Cardiovascular or respiratory conditions	29.9	31.6	28.1

Anxiety or depressive disorders	14.5	12.5	14.6

Sleeping disorders	4.3	3.5	4.6

Digestive disorders	8.0	6.7	8.1

Thirdly, severity of MSD was assessed with the SF-12 instrument, a standardised measure of QOL. The mean scores adjusted for age, sex, co-morbidities and whether the treating physician was the regular physician or not, showed no difference between the types of practices, the mental and physical SF-12 scores being almost identical (Table 4). Comparisons between chronic and non-chronic patients however showed lower SF-12 values indicating poorer QOL in chronic patients in the CM or Mx practice groups but not in the Ho group.

**Table 4:** Quality of life in patients with musculoskeletal disorders (MSD) in three types of primary care practice (N = 1,692)

Type of primary care practice									
	Conventional Medicine			Mixed			Homeopathic		
	**All**	**Acute**^**1**^	**Chronic**^**1**^	**All**	**Acute**	**Chronic**	**All**	**Acute**	**Chronic**

**Quality of life SF-12**									
Mental score ^2^	41.7	43.2*	40.0*	42.0	42.9*	41.1*	41.7	41.2	42.0
Mean (SD)	(10.7)	(9.5)	(11.5)	(10.3)	(10.4)	(10.1)	(9.6)	(9.3)	(9.7)

Physical score ^2^	42.4	43.4	41.3	41.9	43.1*	40.7*	42.8	43.6	42.3
Mean (SD)	(10.4)	(10.3)	(10.4)	(10.4)	(10.9)	(9.8)	(10.8)	(10.1)	(11.2)

1 Chronicity defined as duration of the current MSD episode for more than 12 weeks.

2 Adjusted means in covariance analyses including age, sex, presence of co-morbidities and regular treating physician or not.

* Differences between acute and chronic patients statistically significant at p < 0.05.

## Discussion

This study is one of few providing comparative data on use of homeopathy and other types of CAMs compared to conventional primary care in a representative population of patients. The results showed that patients who consult for MSDs were comparable for quality of life and co-morbidities regardless of the physicians' preferences for prescribing homeopathy or CAMs. This corresponds to what has been reported for physical QOL score but not for mental score where patients using CAMs have been found to have a slightly lower mental scores [5-9]. Our study also showed that patients with chronic MSDs tended to seek care more often with GPs who prescribe alternatives to conventional medicine, a finding that has been reported in other studies [10,11]. Socio-demographic and lifestyle differences between MSD patients in the three groups of physicians could have contributed in part to the results. For instance, patients consulting homeopaths were more often older and more educated women with less lifestyle risk factors than those consulting in conventional primary care. This corresponds to what has been described in other studies of consultations in homeopathy in general [12-15]. However, these factors were controlled for in the analyses and the magnitude of the difference with regard to chronicity was too high to be explained by confounding factors alone.

We also found that these chronic MSD patients more often declared their physician as not being their regular treating physician. In France each citizen is required to identify a physician for their regular health care. Therefore, patients who did not declare their physician as their regular physician, could be considered as consulting in second intention, outside of their regular primary care provider. The greater health care load assumed by physicians who prescribe homeopathy and CAMs then comes from two factors, greater proportions of chronic patients and consultations in second intention. This finding has significant bearing on professional practice as almost half of the homeopath clientele in this study was seen as a consultant. This information provided an important complement to what has been reported on the planning of homeopathic practice, particularly as it differs from conventional medicine [16].

One strength of the study was that MSD patients were identified from a larger survey of patients consulting for any reason in primary care, thus minimising selection bias related to sampling MSD patients directly. Another strength was the specificity of the data collection for the purposes of this study, combining medical information on diagnoses and patients' information on QOL, drugs and CAMs utilisation, all collected on the day of consultation, ensuring timely compatibility with diagnoses. The large number of participating physicians and patients favoured a fair representativity of clinical practices in primary care in France. A previous analysis of the EPI3-LASER survey showed that the distribution of physicians' individual characteristics differed only slightly from published French national statistics [4]. SF-12 scores observed in our patients were also not far away from those reported in three European population surveys of MSD patients, with score differences between acute and chronic patients that were also similar [17,18].

The main limitation of this study was the way the three groups of physicians were defined, relying on their own declaration of prescribing CAMs never or rarely (CM) or regularly (Mx). The definition used for defining the group of homeopaths (Ho) was more straightforward, being based on their professional certification. These definitions potentially limit the generalisation of the results as they represent the practice in France. On the other hand, it also represents a strength because it provided a unique opportunity to compare head-to-head primary care practices differing only by preferences for homeopathy and CAMs, all physicians shared similar medical professional status and basic training in conventional medicine. We feel that even if the context of the study was specific to one country, differences between the groups of patients may provide valid information on the differential utilisation of homeopathy and CAMs, meaningful beyond national borders.

Another important difference with studies performed in other countries is that France is unique with Germany to reimburse homeopathy in a national health insurance regime. Therefore, access to this type of medical practice is specific and, unlike what has been reported in the literature, less subjected to economic barriers [13]. The best illustration of this came from our observation of no apparent differences of access to homeopathy and CAM by employment status. The economic impacts of homeopathy and CAM on health care deserve more attention in future research, particularly in terms of the cost-benefit of complementary approaches for chronic MSD patients who seek alternatives to conventional medicine for the relief of their symptoms.

## Conclusion

MSD patients consulting a primary care physician who prescribed homeopathy and CAMs differed from those seen in conventional medicine. Accounting for the socio-demographic and lifestyle differences, chronic MSD patients represented a greater proportion of the clientele in physicians offering alternatives to conventional medicine. In addition, they treated chronic patients as consulting rather than regular treating physicians, with potentially important impacts upon professional health care practices and organisation.

## Competing interests

The authors declare that they have no competing interests.

## Authors' contributions

All authors are members of the scientific committee that developed and approved the study protocol and the analyses plan, discussed and interpreted the results and revised the manuscript. MR, FL and LA drafted the manuscript, LBG supervised all operational aspects of the study including recruitments, data collection and management. MR supervised data analyses. All authors have read and approved the final manuscript.

## Pre-publication history

The pre-publication history for this paper can be accessed here:

http://www.biomedcentral.com/1471-2474/12/21/prepub
